# New challenges in management of phenylketonuria in pregnancy: a case report

**DOI:** 10.1186/s13256-023-04209-0

**Published:** 2023-11-09

**Authors:** Beatriz Ugalde-Abiega, Sinziana Stanescu, Amaya Belanger, Mercedes Martinez-Pardo, Francisco Arrieta

**Affiliations:** Unidad de Enfermedades Metabólicas, Hospital Universitario Ramón y Cajal, IRYCIS, Crta de Colmenar Viejo, km 9,100, 28034 Madrid, Spain

**Keywords:** Phenylketonuria, Maternal phenylketonuria syndrome, Pregnancy, Nutritional therapy, Case Report

## Abstract

**Background:**

Phenylketonuria (PKU) is an autosomal recessive disease that belongs to a group of disorders resulting from inborn errors of protein metabolism. It was the first disease included in neonatal screening. Neonatal screening has allowed an early diagnosis and treatment of the disease. As a result, an increasing number of women diagnosed with phenylketonuria have reached the reproductive phase of life in good health, and management of pregnancy in women with PKU is becoming more frequent.

**Case presentation:**

In this study, we report the case of a 28-year-old Caucasian patient being followed up for phenylketonuria at Ramón y Cajal Hospital’s Metabolic Diseases Unit. We describe the patient’s gestation, impacted by her and her partner’s diagnosis of PKU, classic and mild phenotypes, respectively, resulting in the fetus affectation.

**Conclusions:**

The description of PKU management—diagnosis, follow-up, and treatment—for both that of patient and that of the gestation with fetus affectation covers a wide sample scenario that shows the effectiveness of pregnancy planning and monitoring of females with PKU and questions the need to carry out a genetic study of gene PKU in the study of fertility.

## Background

Phenylketonuria (PKU) is specifically an aminoacidopathy caused by a defect of the hepatic hydroxylation of phenylalanine (PAH), which results in the accumulation of phenylalanine in the central nervous system. PAH turns phenylalanine into tyrosine, with the reaction involving a coenzyme, namely tetrahydrobiopterin (BH4), as well. Some phenylketonurias are caused by tetrahydrobiopterin synthesis or recycling defects [1]. It is an autosomal recessive disease that belongs to a group of disorders resulting from inborn errors of protein metabolism. More than 500 gene PAH mutations have been reported [2].

With an incidence of 1 in 10,000 live births in Europe and 1 in 15,000 in the USA, PKU is the most common treatable genetic cause of intellectual disability [3].

Phenylketonuria was the first disease included in neonatal screening, allowing for early diagnosis and treatment and the ensuing prevention of neurological involvement. Phenylalanine levels, as well as phenylalanine/tyrosine ratio, are established by means of tandem mass spectrometry from 24 to 72 hours of birth. According to the recommendations of the European and US guidelines, those newborns having phenylalanine plasma levels exceeding 360 μmol/L will undergo treatment.

Enzyme deficiency is variable. There exist different types of phenylketonuria depending on PAH activity function. PAH activity over 10% (benign hyperphenylalaninemia), the patient’s phenylalanine levels in blood ranging from 120 μmol/L to 360 μmol/L, may not require dietary restrictions. However, classic phenylketonuria occurs when PAH activity is under 1% and the patient’s phenylalanine levels are over 1200 mg/dL when diagnosed, which requires strict diet control and protein intake reduction. There are two types between benign and classic phenylketonuria: moderate (PAH 5–10%) and mild (PAH 1–5%) [4].

Neonatal screening has allowed for an early diagnosis of the disease. As a result, an increasing number of women diagnosed with phenylketonuria have reached the reproductive phase of life in good health [5]. When the mother has been previously diagnosed with PKU, the child inherits a minimum of one disease allele. Mothers with PKU have two PAH mutations, whether in homozygous status (two identical mutations) or compound heterozygote (two different mutations). It depends on the father’s carrier state whether the child shows signs of the disorder, as a faulty gene has to be inherited from each parent. A proportion of 1 in 120 will inherit a PAH disease gene from each parent and develop PKU.

Untreated maternal phenylketonuria or hyperphenylalaninemia during pregnancy may lead to maternal phenylketonuria syndrome in the neonate (6, 7). Fetal syndrome by hyperphenylalaninemia is a result of the phenylalanine teratogenic effect on the fetus when phenylalanine levels have not been controlled at gestation. Described by Dent in 1957 and Mabry and Cols in 1963, it causes mental retardation, microcephaly, increased risk of repeated spontaneous miscarriages, intrauterine growth retardation, kidney malformations, and congenital heart disease, mainly malformations in the left chambers, with the frequency of these alterations depending upon the mother’s phenylalanine levels at gestation [6].

Phenylalanine (Phe) transfers across the placenta by means of active transporters, which leads to an increase in phenylalanine levels in the fetus ranging from 70% to 80% over the levels in the mother’s blood. Due to fetal immaturity, the fetus needs the mother’s PAH function to achieve the hydroxylation of phenylalanine, as it is not active until the 26 weeks of pregnancy [8]. Current guidelines recommend maintaining Phe levels between 120 and 360 μmol/L, starting from the preconceptional period [1]. Adequate fetal development depends on strict planning, monitoring, and treatment of phenylalanine levels.

In the present study, we report a case of a 28-year-old patient being followed up for phenylketonuria at Ramón y Cajal Hospital’s Metabolic Diseases Unit. We describe the patient’s gestation, impacted by her and her partner’s diagnosis of phenylketonuria, classic and mild phenotypes, respectively, resulting in the fetus affectation. Given the exceptional nature of the case, we consider its publication of interest.

## Case presentation

We describe a case of a 28-year-old Caucasian patient followed up for phenylketonuria at Ramón y Cajal Hospital’s Metabolic Diseases Unit. The patient was diagnosed with classic PKU, (classic phenotype: L348V/IVS1nt5) at the neonatal screening heel stick test. She had no known family history of phenylketonuria. PKU levels when diagnosed were 1513 μmol/L, and she did not show a positive response to a tetrahydrobiopterin (BH4) loading test. She was treated with 8 g high biological value proteins and 80 g phenylalanine-free protein divided into four daily administrations. Phenylketonuria levels showed to be correct at monthly checks (less than 360 μmol/L) and her adherence to treatment was adequate.

Her partner had also been diagnosed with mild phenotype phenylketonuria (phenotype I65T/R261Q). He was receiving nutritional treatment: high biological value natural proteins, 28 g daily, and 60 g phenylalanine-free protein divided into four daily intakes. He was found responsive to the tetrahydrobiopterin (BH4), so in addition to nutritional treatment, he was also treated with KUVAN with 12 tablets a day. They were not relatives.

**Fig. 1:**
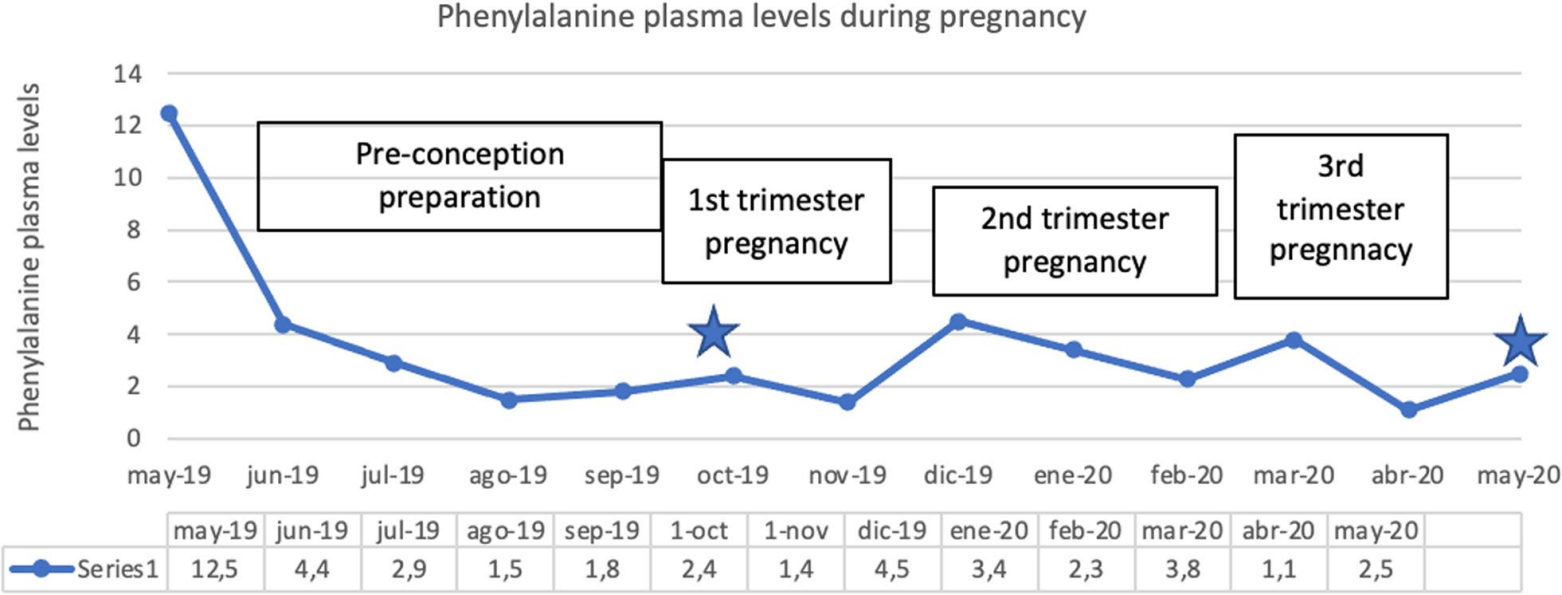
Phenylalanine plasma levels of our patient during pregnancy [8]

In consultation, the patient stated she would like to get pregnant in the following months. We started gestation planning aiming at reducing phenylalanine levels to a range between 120–360 μmol/L for 3 months before pregnancy and kept the same range the whole gestation. The treatment was based on a nutritional therapy to prevent the maternal phenylketonuria syndrome. The treatment included a diet approach which limits high and medium biological value natural proteins to exclude phenylalanine intake. The only proteins allowed were those containing phenylalanine-free essential amino acids such as carbohydrates, essential fatty acids, trace-elements, and vitamins. We prescribed PKU Air 20, 100 g/day divided into five daily intakes, approximately an additional 15% of her protein needs. Low protein foods such as fruit, vegetables, and peeled potatoes, could be freely consumed. A strict follow-up of phenylalanine levels was conducted at our unit every 15 days (Fig. 1).

After several months of adequate control, the patient got pregnant. During gestation, phenylalanine levels were kept smoothly at less than < 360 μmol/L up to the second term, when recurring colds accounted for an increase, which was promptly controlled. The patient was a primary school teacher, and the Gynecology Service gave her medical leave for risky pregnancy from week 18. The patient followed the nutritional pattern closely, there were no pregnancy complications and adequate phenylalanine levels were kept. The fetus doubled maternal phenylalanine levels since fetal hepatic PAH activity was not developed until 26 weeks of pregnancy and the mother was the only metabolic filter for phenylalanine [8]. No changes in diet in our patient were made as the fetus had phenylketonuria and it was not going to improve protein tolerance from week 26 of pregnancy. Follow-up by the Gynecology Service and ultrasound scan controls showed no pathological findings. As in all pregnancies, we monitored the input of nutrients such as folic acid, vitamin B12, iron, ferritin, calcium, and essential fatty acids.

Pregnancy termination occurred in week 34 of gestation with a planned cesarean section for intrauterine growth restriction with vascular redistribution. The baby was delivered with no complications—weight of 2430 kg [−4.3 standard deviation (SD)], height of 45 cm (−4.8 SD), head circumference of 28.6 cm (1.81 SD), and an apgar score of 9/10. The neonatal physical and neurological assessment was normal. The evaluation of the newborn’s level of alertness, cranial nerve function, motor and sensory system function, and primitive reflexes revealed no abnormalities.

The examination of the development milestones of the infant was normal. At 3 months [weight of 5.005 kg (−1.33 SD), height of 55.5 cm (−1.8 SD), head circumference of 38.4 cm (1.81 SD)], head support was stable, tone and spontaneous mobility were normal. The infant was able to follow objects with his eyes, laugh, and hold things in both hands. At 9 months [weight of 9.60 kg (2.4 SD), height of 73.72 cm (2 SD), head circumference of 43.5 cm (−1.5 SD)], the infant babbled and could stand with support, and at 10 months, he was able to walk with support. At 12 months, the psychomotor development of the child was normal.

From birth, the baby had a diet including closely monitored protein intake. It was diagnosed with phenylketonuria, phenotype moderate to severe. However, phenylalanine levels could not be assessed when diagnosed due to the diet started after birth. For the first months, the baby was treated with KUVAN (BH4), but, since tolerance to treatment was not achieved, it was discontinued. After suspension, no changes were found in the metabolic control and the baby was not considered to be BH4 responsive.

## Discussion and conclusions

The case reported allows for the description of PKU management at our Ramón y Cajal Hospital’s Metabolic Diseases Unit—including diagnosis, follow-up, and treatment—moving from the context of a single patient to that of gestation with fetus affectation.

We present the case specifics stating the main guidelines of management to provide a clinical scenario, which we are increasingly bound to encounter.

Mutations in the *PAH* gene cause PKU, which causes low levels of phenylalanine hydroxylase. As a result, phenylalanine from a person’s diet cannot be metabolized, thus building up to toxic levels, which causes mental disability and other serious problems. The process can be controlled with adequate diagnosis and follow-up, the basis of PKU treatment being a strict lifelong diet with low phenylalanine. The goal of the treatment is the prevention of accumulation of excessive phenylalanine by the control of natural protein intake and replacement of natural protein by phenylalanine-free proteins [9].

Firstly, it is important to highlight that blood phenylalanine levels provide a reliable method for the diagnosis and monitoring of the metabolic status of the patient. Our patient had been diagnosed at the neonatal screening heel stick test, not showing a positive response to a tetrahydrobiopterin (BH4) loading test. She was treated with 8 g high biological value proteins and 80 g phenylalanine-free protein, her phenylalanine levels showed to be correct at monthly checks and her adherence to treatment was adequate.

Secondly, as PKU is an autosomal recessive disorder, a baby will have the disease when they inherit a mutated copy of the disease-causing gene from each parent. When the mother has been diagnosed, it is the genetic status of the father that determines the child’s risk of having the disease. Our case is especially illustrative in this respect as well, since both parents had PKU, which led to fetus affectation. Besides nutritional treatment, including high biological value natural proteins, 28 g daily, and 60 g phenylalanine-free protein, the father was on KUVAN (BH4) after having been found responsive to the tetrahydrobiopterin (BH4) loading test.

Thirdly, moving to the patient’s gestation, the management of our case shows the effectiveness of strict planning, monitoring of phenylalanine levels, and treatment to ensure adequate fetal development. The reported intervention proves effective to prevent the fetal syndrome by hyperphenylalaninemia, a result of the phenylalanine teratogenic effect on the fetus when phenylalanine levels have not been controlled at gestation. Prick *et al.*, who published a large cohort of pregnant women diagnosed with PKU without treatment, underline that treatment during pregnancy is of great importance to prevent neonatal sequelae [6].

Rohde *et al.* have described in their studies the need for the development of training programs by specialized metabolic centers for females with PKU at childbearing ages since those mothers are at risk for giving birth to a child with maternal PKU syndrome, and, on many occasions, they are unaware of their suboptimal metabolic control [10]. These programs should involve in the training of not only the patients themselves, but also their partners and family members.

When our patient expressed her wish to get pregnant, gestation planning aiming at reducing phenylalanine levels to a range between 120–360 μmol/L was started. To prevent the maternal phenylketonuria syndrome, she was put on a diet [7] that limited high and medium biological value natural proteins [9] and was prescribed PKU Air 20, 100 g/day over five daily intakes, approximately an additional 15% of her protein needs. Her phenylalanine levels were monitored every 15 days. The improvement of phenylalanine tolerance from 20 weeks of gestation, because of increased fetal phenylalanine hydroxylase activity [6], ensures an increase in the contribution of phenylalanine and a progressively more normalized diet. No changes were made in our patient’s diet throughout pregnancy as the baby would be unable to carry out the hydroxylation of phenylalanine into tyrosine and would be affected with PKU since both parents had the disease.

The importance of controlling phenylalanine levels and controlling them as soon as they are detected is more than described in different studies [6, 11, 12]. The incidence of microcephaly increases by 5–18% when control is not attained by week 10 of gestation and increases up to 67% if control is not achieved before week 30 of gestation. Risks for congenital heart disease may also be increased in individuals due to poor protein intake and vitamin B12 deficiency [7]. The incidence of intrauterine growth retardation is not increased if Phe control is achieved by 10 weeks’ gestation; however, it increases with later onset of Phe control [13]. Therefore, unplanned pregnancies should be strictly avoided.

After birth, diagnostic methods prove essential, as well. In the newborn screening PKU test, the infant is tested for the disorder in the first days of life while the tetrahydrobiopterin (BH4) loading test assesses response and determines subsequent treatment. To prevent untreated pregnancies via detecting undiagnosed adults, countries where a significant number of women of childbearing age were not screened as newborns may consider prepregnancy PKU screening [12].

In our case, the baby was diagnosed with phenylketonuria, phenotype moderate to severe, but it was not possible to assess phenylalanine levels at diagnosis as a closely monitored protein intake diet had been started after birth. Treatment with KUVAN was discontinued after the first months, due to lack of tolerance with no changes in the metabolic control. The baby was not found to be BH4 responsive.

## Conclusion

The search of PKU in neonates through newborn screening has proved to be an effective procedure in the prevention of the maternal phenylketonuria syndrome. The infants diagnosed with PKU, monitored, and treated at pediatrics services, and transitioned to endocrinology units on reaching adult age, have new needs that we should be prepared to meet. Since females with PKU in childbearing age are at risk of giving birth to a child with the syndrome, they should be especially aware of the importance of their metabolic control, and of planning and monitoring their pregnancies.

The situation in the case reported is becoming increasingly more common in our clinical practice. The description of PKU management, including diagnosis, follow-up, and treatment, in both that of the patient and that the of gestation with fetus affectation, covers a wide sample scenario that shows the effectiveness of pregnancy planning and monitoring of females with PKU and questions the need to carry out a genetic study of gene PKU in study of fertility.

## Data Availability

If supporting data is needed contact the correspondence author.
